# GEO uploader: simplifying the data deposition in the GEO repository

**DOI:** 10.1186/s12859-026-06466-4

**Published:** 2026-05-05

**Authors:** Ronald Domi, Falko Noé, Peter Leary, Hubert Rehrauer

**Affiliations:** 1 Functional Genomics Center Zurich, ETH Zurich and University of Zurich, Zurich, Switzerland; 2 Swiss Institute of Bioinformatics, Lausanne, Switzerland

**Keywords:** Research data management, Open science, Genomics data sharing, Submission automation, Bioinformatics workflow, Scientific data repositories, Reproducible research

## Abstract

**Background:**

The Gene Expression Omnibus (GEO) (Clough and Barrett in: methods in molecular biology, Clifton, 2016) repository requires complex multistep submissions involving metadata preparation, FTP uploads, and MD5 validation. Current manual processes are error-prone, time-consuming, and require significant bioinformatics expertise, creating barriers for many researchers.

**Results:**

We present GEO Uploader, a web-based tool that automates the entire GEO submission workflow through an intuitive interface. The application reduces the submission initiation time from 2–3 h to under 20 s by automating file uploads, MD5 calculations, and metadata template population. Key features include parallel processing of uploads and checksum calculations, automated error prevention through template-based metadata completion, real-time progress tracking, and support for complex submission structures. Deployment across 30 + users with 50 + upload sessions, including datasets exceeding hundreds of gigabytes, demonstrates practical utility and reliability in research environments.

**Conclusion:**

GEO Uploader significantly reduces the technical barrier for GEO submissions while minimizing errors through comprehensive automation. The tool supports data sharing by enabling researchers without specialized bioinformatics expertise to complete submissions independently. Available as open-source software with multiuser deployment capabilities, GEO Uploader represents a substantial improvement in research data sharing accessibility and supports broader adoption of open science practices in the genomics community.

## Background

The Gene Expression Omnibus (GEO) serves as the primary public repository for functional genomics data, hosting most datasets generated by the research community. To comply with data sharing mandates from major life science journals, researchers are required to deposit their data in a public repository that follows the FAIR principles of data sharing. For sequencing- and array-based transcriptomics data, GEO is a popular choice [2], and at the time of publication, the GEO repository contained 8 million samples from more than 250,000 studies [3], highlighting its essential role in supporting reproducible research and enabling data reuse.

Despite its importance, submitting data to GEO involves complex technical steps and can be time-consuming, posing significant barriers for many researchers. A two-week preparation period is recommended prior to manuscript submission, reserving one week for data organization and metadata preparation, five business days for quality control and validation, and additional time for potential resubmission if errors are encountered. GEO’s three-step submission workflow is as follows: (1) organize files and upload them to GEO servers via FTP, (2) generate MD5 checksums for data integrity verification, and (3) complete the structured Metadata.xlsx template to link uploaded files with experimental samples. Errors at any stage result in submission rejection, which can delay publication and add to the workload of researchers.

Current solutions address only partial aspects of the submission workflow, creating fragmented user experiences that still require significant manual intervention. Available tools generally target isolated tasks, such as MD5 checksum generation or file transfer, and are implemented primarily as command-line utilities requiring Bash proficiency. For example, tools such as *GREIN* [4] have enhanced GEO data analysis and visualization capabilities, but the critical upload functionality remains unaddressed. *geo_prepper* [5] provides automated metadata formatting but lacks transfer capabilities. The *GEO-submission guide* [6] offers comprehensive documentation and sample templates but still necessitates manual adaptation for each study’s specific requirements and provides no automated validation of metadata completeness or formatting.

Manually combining outputs from separate tools into the metadata template introduces data entry errors and reinforces the technical challenges faced by nonexpert users. The challenges become evident in paired-end RNA-seq studies containing 24 samples, which require uploading 48 + files (raw and processed data) while completing over 15 metadata fields per sample, creating substantial potential for human error.

The technical complexity of current solutions limits GEO submissions to users with strong computational skills, limiting broader community participation. To address this gap, we developed GEO Uploader, an integrated web-based platform that automates the entire submission workflow while providing an accessible interface for researchers regardless of their computational background. The tool highlights error prevention by automating metadata entry and checking for completeness, eliminating common sources of submission failure while significantly reducing the time investment required for successful data deposition.

## Implementation

**Fig. 1:**
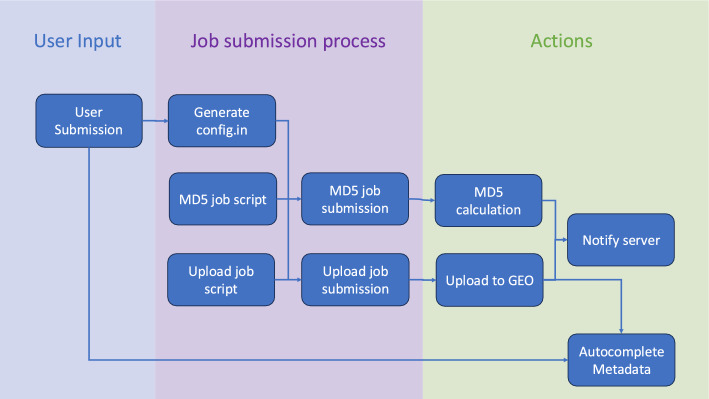
This illustration outlines the complete GEO Uploader workflow automation process. Upon user submission through the web interface, the system performs three parallel operations: upload job creation, MD5 checksum job creation, and metadata template updating. Using the generated configuration file containing the list of files to upload and FTP credentials, the tool submits the tasks to the job scheduler for background processing. Two parallel Python modules execute simultaneously: file upload to GEO servers and MD5 checksum generation with output to md5sheet.tsv. Upon completion of both processes, the server receives a notification and marks the submission as complete, allowing users to download the populated metadata template for the final GEO submission

GEO Uploader is implemented as a web application via the Python Flask framework, offering compatibility with the Mac, Linux, and Windows systems. The application uses a modular architecture, separating backend services into core and external components (Fig. 1). Core services manage the main application logic, whereas external services handle tasks such as job submission, user authentication, and directory management. This separation allows the tool to adapt to different deployment scenarios: External services can integrate with existing facility infrastructure (such as institutional job schedulers and authentication systems) for centralized deployments or use default implementations for standalone installations on a client computer.

The frontend uses HTML with Jinja2 templating, Bootstrap for responsive design, and JavaScript for interactive elements. Installation and configuration details are maintained in the project’s GitHub repository, with Conda or Mamba package managers as the sole requirement for setup.

The application treats each GEO submission as a separate project, creating and managing dedicated artifacts for each one. This ensures that all files and data related to a submission are organized and stored independently. Upon submission creation, the system generates a unique artifact folder containing all submission-related components: File lists for upload, FTP connection parameters, MD5 calculation jobs, and comprehensive job execution logs.

Users start a submission by entering their FTP credentials for their assigned GEO space and choosing the specific files they want to upload. The system accepts any file format without imposed restrictions, giving users complete discretion over submission content. No file size limitations or format validation rules are enforced, maintaining flexibility for diverse data types and experimental approaches.

Metadata sheet generation uses a centralized template system within the codebase. Each new submission creates a copy of the master metadata template, which is then populated with submission-specific information, including file names, MD5 checksums, and user-defined sample assignments. One limitation of this approach is that when GEO updates the metadata sheet format, it becomes the responsibility of the maintainer to update the local template copy.

The web interface displays the metadata sheet for users to complete, with file names and technical parameters prefilled to reduce typing errors. Users are responsible for completing study descriptions, sample information, and protocol details through the web form interface.

Upon user confirmation, GEO Uploader launches two parallel processes: MD5 checksum calculation and file upload operations. The MD5 job computes hashes for selected files and populates the corresponding columns in the metadata template. The upload job transfers files to the FTP server one at a time in a sequential manner, utilizing a single connection approach.

Real-time progress tracking provides users with detailed visibility into submission status. The monitoring pages display already processed files, the remaining queue items, and the current job status. Job logs are accessible through the artifact folder created on the file system.

Upon completion of file uploads and MD5 calculations, users can download the completed metadata sheet for final submission to GEO through the standard web interface.

### Core facility integration

**Fig. 2:**
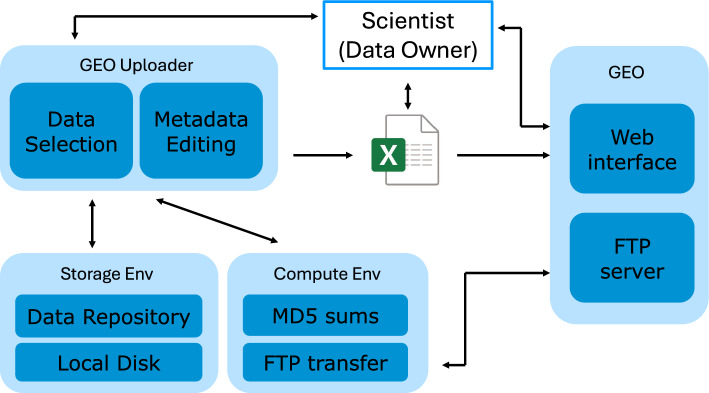
Visual representation of the different components. The diagram illustrates how the GEO Uploader acts as an intermediary layer, separating the data owner from both the storage environment and the compute environment by managing all computational processes on their behalf

GEO Uploader is primarily designed for deployment in core facilities, while still supporting individual end-users as a localhost web application for local files upload. The schematic process is shown in Fig. 2. In a core facility setting, the uploader can be integrated with existing authentication and file management systems, making sample collection, file assignment, and folder exploration seamless. The GEO Uploader can load sample meta-information from a structured tabular file. By relying on the facility’s infrastructure—such as LDAP, SLURM, fileserver—the system can allow registered users to upload using a scheduler for resource isolation.

The GEO Uploader is in operation at the Functional Genomics Center Zurich and integrates with SUSHI [7] and B-Fabric [8].

## Results and discussions

GEO Uploader demonstrates performance improvements over manual submission processes. Prior to implementation, bioinformaticians required 2–3 h to manually prepare submissions, including file organization, MD5 checksum calculation, and metadata completion. Manual processes were prone to data entry errors during file listing and checksum entry, sometimes leading to resubmission cycles that further extended timelines.

GEO Uploader reduces the submission launch time to approximately 20 s, after which all processing is handled by background jobs. This allows bioinformaticians to focus on other tasks without needing to monitor or manually manage the submission process. The elimination of checksum calculations and manual file handling removes the primary sources of human error that previously caused submission delays.

A significant practical improvement addresses the network connectivity issues that affect manual FTP uploads. Users commonly encounter connection timeouts during manual transfers, requiring session restarts. GEO Uploader’s Python-based FTP libraries provide more robust connection handling, eliminating the timeout problems that made manual submissions unreliable and time-consuming.

Most users successfully complete submissions after understanding how GEO uploads work, so we integrate links to a help page [9] into the web interface to enhance the onboarding experience and better explain the submission requirements to new users.

**Fig. 3:**
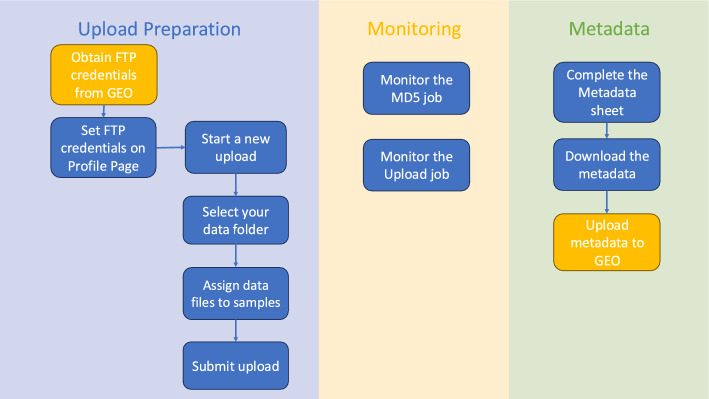
This illustration outlines the complete user workflow for GEO submission. Users must first obtain their personal GEO FTP credentials by following the helper wiki [9] and saving them to their profile page. Once credentials are saved, users can initiate a new submission by selecting files for upload and submitting the form, which then triggers background processing jobs. All job progress can be tracked through individual dashboard pages. Users can begin preparing their metadata sheet immediately and then download and submit the completed metadata to GEO once the MD5 verification job has finished

One notable advantage of the automated approach (Fig. 3) is improved handling of complex, heterogeneous submissions that were previously impractical to manage manually. The "long tail" of submissions involving complicated file structures, mixed data types, or large numbers of files can now be processed systematically without the manual overhead that makes such submissions overly time-consuming. This capability expands the practical scope of data sharing for complex experimental designs.

Since deployment, the tool has processed over 50 upload sessions across more than 30 users, including non-bioinformaticians, handling datasets ranging from standard RNA-seq studies to large-scale submissions exceeding 500 GB. Multiuser deployment with session isolation has proven effective for institutional use, allowing multiple researchers to work simultaneously without interference.

Current limitations include the lack of automatic resubmission when upload errors occur, which requires manual intervention for failed transfers. Additionally, the final metadata submission still requires users to access the GEO website directly, presenting an opportunity for further integration. The tool also requires ongoing maintenance to synchronize with the GEO metadata template updates. When GEO modifies the required metadata structure, column requirements, or dropdown options, the local template must be updated accordingly. Authentication functionality, while implemented for multiuser deployments, adds unnecessary complexity for single-user installations. The modular job service architecture allows replacement with alternative background processing systems better suited to specific deployment environments such as Slurm.

## Conclusion

GEO Uploader addresses a critical bottleneck in genomics research by automating the complex and error-prone process of data submission to the Gene Expression Omnibus repository. By reducing the submission preparation time from 2–3 h to under 20 s and eliminating common data entry errors through automated file handling and metadata population, the tool significantly lowers the barrier to data sharing compliance required by major scientific journals.

The web-based interface successfully supports GEO submissions, enabling researchers without specialized bioinformatics expertise to complete uploads independently. The automation of technical components, including MD5 checksum calculations, FTP upload management, and metadata template completion, provides almost end-to-end support for this upload. This capability is particularly valuable for the "long tail" of submissions involving heterogeneous file structures that were previously impractical to manage manually.

GEO Uploader reduces technical barriers for uploading functional genomics data to GEO and therefore supports the broader goals of reproducible research and data reuse in the genomics community.

Project name: GEO Uploader.

Project home page: https://github.com/fgcz/geo-uploader

Operating system(s): Platform independent.

Programming language: Python, HTML, Javascript.

Other requirements: Docker.

License: MIT.

Any restrictions to use by nonacademics: None.

## Data Availability

GEO Uploader is available on github at https://github.com/fgcz/geo-uploader. The repository contains the source code, as well as a tutorial, documentation and synthetic example data.

## References

[1] Clough E, Barrett T. The gene expression omnibus database. In: methods in molecular biology (Clifton, N.J.) 2016, 1418. 10.1007/978-1-4939-3578-9_5 PMC4944384 27008011

[2] Reporting standards and availability of data, materials, code and protocols [https://www.nature.com/nature-portfolio/editorial-policies/reporting-standards#mandates-for-specific-datasets].

[3] Home - GEO - NCBI [https://www.ncbi.nlm.nih.gov/geo/].

[4] GitHub - uc-bd2k/GREIN: GREIN : GEO RNA-seq experiments interactive navigator [https://github.com/uc-bd2k/GREIN].

[5] GitHub - NICHD-BSPC/geo_prepper: tool to help prepare data for GEO submission [https://github.com/NICHD-BSPC/geo_prepper].

[6] GitHub - CBMR-single-cell-omics-platform/GEO-submission-guide: guidelines and helper scripts for preparing sequencing data for submission to NCBI GEO [https://github.com/CBMR-Single-Cell-Omics-Platform/GEO-submission-guide].

[7] Hatakeyama M, Opitz L, Russo G, Qi W, Schlapbach R, Rehrauer H. SUSHI: an exquisite recipe for fully documented, reproducible and reusable NGS data analysis. BMC Bioinformatics. 2016;17:1–9. 27255077 10.1186/s12859-016-1104-8 PMC4890512

[8] B-Fabric [https://dl.acm.org/doi/10.1145/1739041.1739135].

[9] geo-uploader/documentation/GEO_instructions.md at main · fgcz/geo-uploader [https://github.com/fgcz/geo-uploader/blob/main/documentation/GEO_instructions.md].

